# Providers’ Experiences Implementing Cognitive Care Planning: Barriers and Facilitators

**DOI:** 10.1002/alz.092477

**Published:** 2025-01-09

**Authors:** Katherine Carroll Britt, Xaviera Xiao, Kevin H Sun, Bin Huang

**Affiliations:** ^1^ the University of Pennsylvania, Philadelphia, PA USA; ^2^ BrainCheck Inc, Austin, TX USA; ^3^ BrainCheck, Inc, HOUSTON, TX USA; ^4^ BrainCheck, Inc, Austin, TX USA

## Abstract

**Background:**

Cognitive care planning—the process of regularly and systematically assessing patient needs and documenting recommendations to address them—improves health and quality of life among patients with cognitive impairments, like Alzheimer’s disease and related dementias (ADRD). A cognitive care plan may promote physical exercise, social engagement, healthier eating, medication recommendations, and overall improvement in care management (e.g., advance care planning), which translates to lower health facility use and better quality of life. As dementia is progressive, even short‐term improvements in quality of life, emotional health, and resource use can significantly alleviate the disease burden. With limited pharmaceutical treatments, cognitive care planning is likely to remain one of the most effective ways to promote care among patients with ADRD. Despite significant potential benefits for patients and caregivers, formal and individualized cognitive care planning remains underutilized.

**Method:**

We conducted a qualitative study exploring providers’ perceptions and experiences implementing cognitive care planning in their practice. A purposive sample of 8 primary care providers actively caring for older adults including patients with ADRD participated in semi‐structured teleconference interviews. Iterative inductive content analysis was used to analyze data.

**Result:**

Our preliminary results indicate several barriers and facilitators for implementing cognitive care planning into practice by providers. Providers reported some familiarity with general cognitive care and assessment but lacked a clear systematic approach and thorough understanding of the cognitive care planning process and steps. Across patient‐ and caregiver‐related, organization‐related, clinical care team‐related, and provider‐related focused areas, we identified five categories: (1) perceptions (buy‐in or reluctance), (2) family presence (context, support, communication, provider‐patient relationship), (3) logistics and priorities (time, schedule, staffing, priorities), (4) structure/systemized approach (standardization, templates, clear roles, referrals), and (5) experience and training (confidence or unfamiliarity).

**Conclusion:**

Providers expressed the value of a structured approach for successfully implementing cognitive care planning. Buy‐in at multiple levels (patient, caregiver, organization, and clinical team) may prompt prioritizing this care approach in practice to restructure the assessment process for older adults. In addition, caregiver and family accompaniment to patient visits can increase contextual knowledge and provider communication for tailoring cognitive care plans.

